# WeChat-Based HIV e-Report, a New Approach for HIV Serostatus Requests and Disclosures Among Men Who Have Sex With Men: Prospective Subgroup Analysis of a Randomized Controlled Trial

**DOI:** 10.2196/44513

**Published:** 2023-05-08

**Authors:** Hai-Tong Sun, Xiao-Ru Fan, Yu-Zhou Gu, Yong-Heng Lu, Jia-Ling Qiu, Qing-Ling Yang, Jing-Hua Li, Jing Gu, Chun Hao

**Affiliations:** 1 Department of Medical Statistics, School of Public Health, Sun Yat-Sen University Guangzhou China; 2 Sun Yat‑Sen Global Health Institute, Institute of State Governance, Sun Yat-Sen University Guangzhou China; 3 Guangzhou Center for Disease Control and Prevention Guangzhou China; 4 Lingnan Community Support Center Guangzhou China

**Keywords:** behavioral intervention, HIV serostatus disclosure, HIV testing, men who have sex with men, mHealth

## Abstract

**Background:**

Requesting and disclosing HIV serostatus is associated with a reduction in HIV transmission among men who have sex with men (MSM). However, the reliability of common methods for HIV serostatus request and disclosure is inadequate. Validated approaches for requesting and disclosing HIV serostatus are necessary.

**Objective:**

The objective of this study was to investigate the use of the HIV e-report as authentic evidence of HIV serostatus among the MSM community in Guangzhou, China. Additionally, the study aimed to explore its correlation with HIV serostatus requesting and disclosure receiving behavior.

**Methods:**

This study is a subgroup analysis of a cluster randomized controlled trial (RCT) that enrolled 357 participants during the first year. Participants in this RCT were recruited from the WeChat-based HIV testing service miniprogram developed by Guangzhou Center for Disease Control and Prevention, China. Participants completed web-based questionnaires at baseline and at the month 3 follow-up, which covered sociodemographic characteristics, HIV-related, HIV serostatus requests, receiving HIV serostatus disclosures, and HIV e-report usage.

**Results:**

The WeChat-based HIV e-report was available in Guangzhou when the RCT project started. At the month 3 follow-up, 32.2% (115/357) of participants had their own HIV e-reports, and 37.8% (135/357) of them had received others’ HIV e-reports. In all, 13.1% (27/205) and 10.5% (16/153) of participants started to use HIV e-reports to request the HIV serostatus from regular and casual male sex partners, respectively. Moreover, 27.3% (42/154) and 16.5% (18/109) of the regular and casual male sex partners, respectively, chose HIV e-reports to disclose their HIV serostatus. Compared to MSM who did not have HIV e-reports, those who had HIV e-reports and stated, “I had had my own HIV e-report(s) but hadn’t sent to others” (multivariate odds ratio 2.71, 95% CI 1.19-6.86; *P*=.02) and “I had had my own HIV e-reports and had sent to others” (multivariate odds ratio 2.67, 95% CI 1.07-7.73; *P*=.048) were more likely to request HIV serostatus from their partners. However, no factor was associated with receiving an HIV serostatus disclosure from partners.

**Conclusions:**

The HIV e-report has been accepted by the MSM community in Guangzhou and could be applied as a new optional approach for HIV serostatus requests and disclosures. This innovative intervention could be effective in promoting infectious disease serostatus disclosure among the related high-risk population.

**Trial Registration:**

ClinicalTrials.gov NCT03984136; https://clinicaltrials.gov/show/NCT03984136

**International Registered Report Identifier (IRRID):**

RR2-10.1186/s12879-021-06484-y

## Introduction

Men who have sex with men (MSM) are a population bearing a disproportionate burden of HIV infection in China [1]. It accounted for 25.5% of new HIV infections [1], and the prevalence in China reached 6% in 2020 [2]. Having unprotected anal intercourse with partners of unknown HIV status accelerates the rising rates of HIV among this population. To prevent HIV acquisition and transmission in the context of condomless sex, it is crucial that MSM are aware of their own and their partner’s HIV status [3]. Such awareness necessitates frequent HIV testing and mutual HIV status disclosure before engaging in condomless sex with partners [4].

Studies indicated that over half of MSM used verbal communication and guessing for HIV serostatus request and disclosure among MSM [5,6]. While taking HIV tests together is a reliable approach to confirm partners’HIV status, some individuals doubt the reliability of HIV self-testing, and self-test kits may not always be readily available. Deception of HIV serostatus in verbal information is prevalent [7] and difficult to confirm. Trusting partners without reliable evidence will lead to an increased risk of HIV infection [6]. It is crucial for MSM to engage in more verified HIV serostatus disclosure before sexual activity, as this can effectively reduce the risk of HIV infection. However, current methods of disclosure are insufficient in meeting this need, indicating a need for more effective strategies.

WeChat, a popular social media app with over 1.2 billion active users [8], similar to Twitter or the mix of WhatsApp and Facebook, isa ubiquitous daily-use app in China [9]. WeChat miniprograms are subapps within the WeChat ecosystem. It has great potential for health intervention research [10]. In Guangzhou city, a unique and well-established WeChat miniprogram of the HIV testing service system in China has been developed by Guangzhou Centers for Disease Control and Prevention (CDC) and the MSM community-based organization Lingnan Partners Community Support Center (hereinafter called “Lingnan Center”) [11]. This miniprogram served web-based HIV testing service appointments, web-based-to-offline referral, offline clinic testing, and HIV results e-report delivery after midyear 2019 coinciding with the start of this research project. The HIV result e-report is convenient and well-reserved on the internet. It is an MSM community demand-driven tool which is codeveloped by CDC and Lingnan Center. Due to the use of digital technology, an HIV e-report cannot be forged, ensuring the CDC authenticity of the serostatus results.

Regular or exchangeable HIV e-reports were applied as an intervention in our whole randomized controlled study [12]. In the context of social exchange theory [13], which posits that individuals engage in rational, reciprocal, and fair exchanges in order to achieve desired outcomes, MSM engage in HIV serostatus disclosure as a means of promoting safe sex. By disclosing their own HIV serostatus or requesting their partner’s status, these principles promote mutual disclosure. If a partner’s HIV serostatus is unknown, the desire for sexual activity may drive MSM to autonomously test for HIV, thus promoting HIV testing behavior.

The objective of this study is to describe the usage of the HIV e-report after it was available in Guangzhou and investigate whether it is associated with promoting HIV serostatus requests and disclosure-related behaviors among this high-risk population.

## Methods

### Study Design

**Figure 1:**
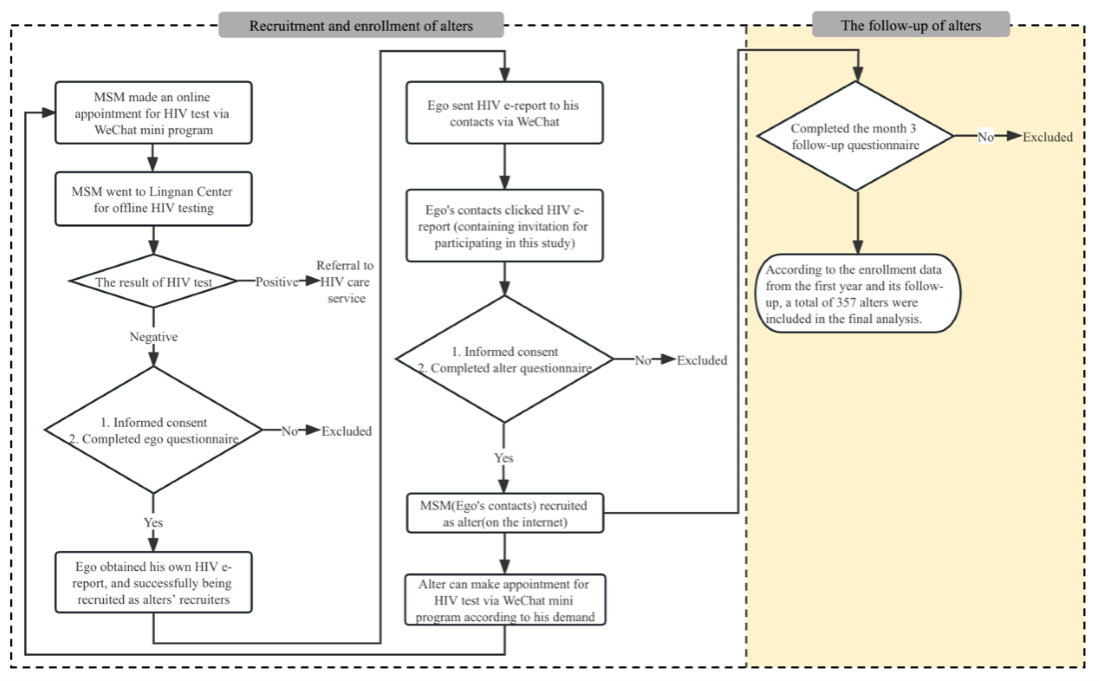
The recruitment and follow-up of alters. MSM: men who have sex with men.

This study is an early-stage subgroup analysis of a cluster randomized controlled trial that was conducted based on the WeChat miniprogram of HIV testing service system in Guangzhou, China (see Figure 1). Guangzhou, the capital city of Guangdong province, where some headquarters of high-tech companies sit, such as Tencent, Huawei, etc, is a megacity with 18 million population in China [14]. On account of the booming economy and tolerant culture toward homosexuality, the earliest internet-based MSM dating platform in China was established in Guangzhou in 1998 [15]. The prevalence of HIV infection in Guangzhou reached 8.27% [16]. This randomized controlled trial aims to increase HIV testing behaviors by using an HIV e-report exchange mechanism among MSM. Further information on the project can be found elsewhere [12].

MSM who were enrolled in the study at the first year and completed the month 3 follow-up were included in the analysis of this study. The HIV e-report was available to MSM in Guangzhou at the beginning of this study.

### Recruitment of Participants

The egocentric social network method [17] which involves participants nominating individuals from their social network to participate in a study, was used to recruit participants for this study. Egos were asked to nominate individuals from their social network (alters) to participate. These alters were then recruited to participate in the study.

**Figure 2:**
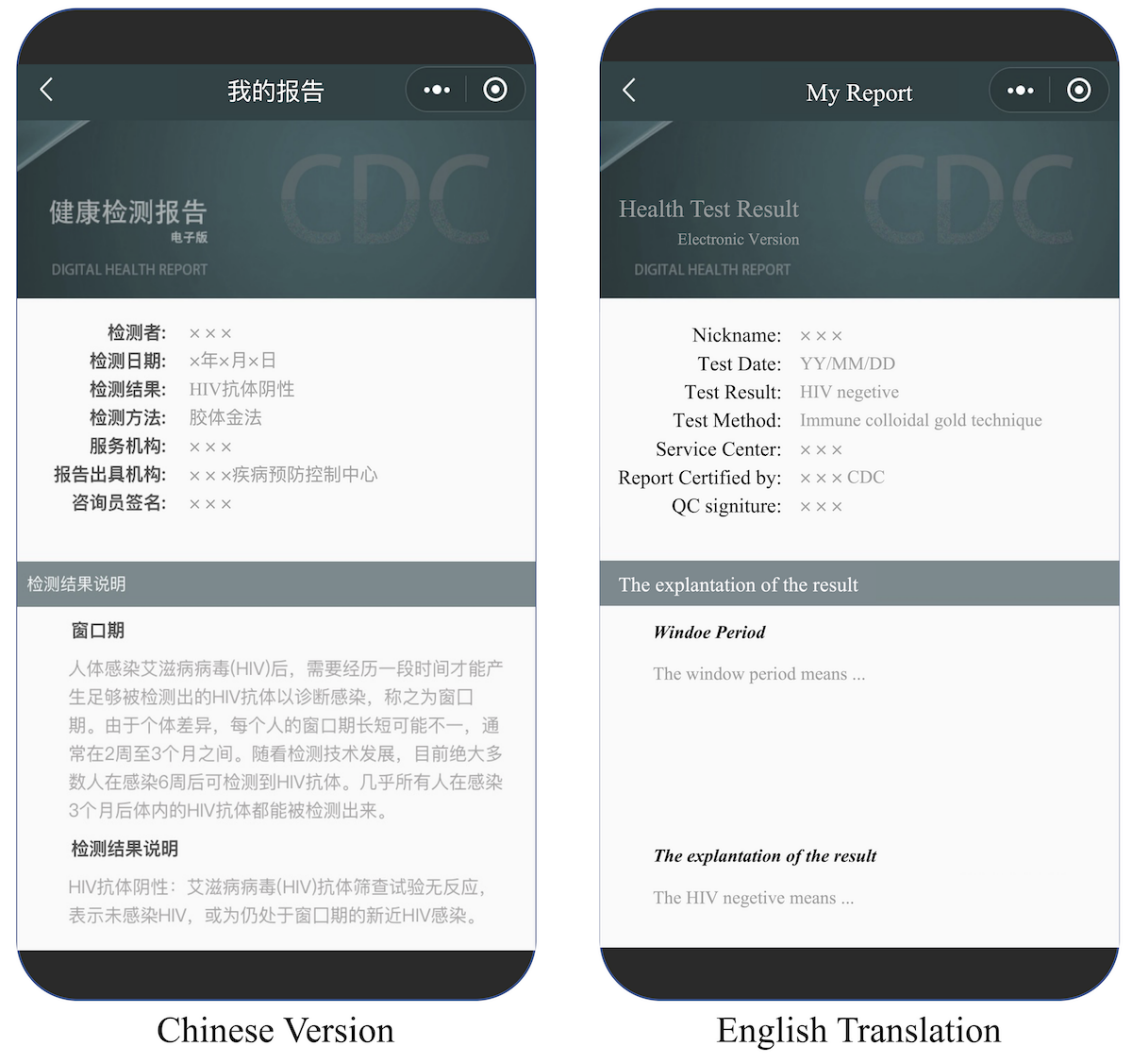
The WeChat-based HIV testing service system (smartphone-based HIV test results report module). The Chinese version image depicted in this figure is the screenshot of the smartphone-based HIV test result report in the WeChat miniprogram. Only Chinese version is available.

Figure 1 shows the progress of recruitment. People who went to Lingnan Center to take HIV antibody tests were recruited as “Egos.” Lingnan Center is an MSM-friendly clinic which is cooperated with Guangzhou CDC. In 2022, 10,292 HIV tests have been done in Guangzhou among MSM, and over 60% of HIV tests among MSM in Guangzhou are conducted by this clinic [11]. Every ego who tested for HIV at Lingnan Center got his own CDC-certified web-based HIV results report, hereinafter referred to as “HIV e-report.” Only MSM who went to Lingnan Center for offline HIV testing can get HIV e-reports. The HIV e-report includes the basic testing information and the test result, which is certified by Guangzhou CDC (see Figure 2). Egos nominated alters by sending HIV e-reports to their contacts via WeChat.

HIV e-report receivers were invited to complete the baseline questionnaire (the link to the questionnaire was attached at the bottom of the HIV e-report) and were recruited as “alters” participants. After 3 months, alters would receive WeChat messages which contain the link to the follow-up questionnaire. Once alters went to Lingnan Center to take HIV antibody tests within 3 months, they would get their own HIV e-reports.

Egos were included in the study if they met the following inclusion criteria: (1) were 18 years of age or older, (2) engaged in anal sex with men, and (3) had confirmed negative HIV status. Alters were included in the study if they met the following inclusion criteria: (1) were 18 years of age or older, (2) were engaged in anal sex with men, (3) planned to reside in Guangzhou for the next year, and (4) had unknown or confirmed negative HIV status. Individuals who were unable to complete the questionnaire for any reason were excluded from the study.

### Measures

#### Sociodemographic Characteristics

All background characteristics of alters were collected in the baseline questionnaire.

Sociodemographic characteristics collected include age, marital status, local residence, length of stay in Guangzhou, education, and income. Participants were asked about MSM-related characteristics, including sex orientation, sex role, and whether to recruit male sex partners on the internet. We dichotomized age by 25 years and income by 5000 RMB (US $700) according to the median.

#### HIV-Related Information

HIV-related information was collected at the month 3 follow-up questionnaire. Participants were asked whether they had intervened by any HIV-related programs and whether they had taken up HIV antibody testing and drug use during sex in the past 3 months.

The risk perception of HIV infection was evaluated with 2 questions. One is “How do you think the prevalence of HIV infection in MSM in Guangzhou.” Considering the prevalence of HIV infection in Guangzhou at 8.27% [16], responses options are “≥10%” and “<10%” as high perceived risk to HIV infection or not. The other is, “Is someone close to you infected with HIV.”

HIV stigma was measured using a 7-item version of the HIV stigma scale [18], designed to measure the extent to which participants anticipated negative interpersonal and intrapersonal consequences were they to contract HIV in the future. All 7 items were rated on a Likert-type scale (1=Strongly Disagree and 4=Strongly Agree). Higher scores are indicative of greater perceived HIV-related stigma. The Cronbach α for HIV stigma was .728.

HIV testing social norms [19] were calculated using 3 survey items rated on a 4-point Likert scale in the web-based survey. All 3 items were rated on a Likert-type scale (1=Strongly Disagree and 4=Strongly Agree). Higher values indicate positive HIV testing social norms. The Cronbach α for HIV testing social norms was .737.

#### HIV Serostatus Request and Disclosure Receiving From Different Kinds of Male Sex Partners

Participants were asked for information about HIV serostatus in the past 3 months from regular male sex partners and casual male sex partners, respectively. Moreover, we used “or” to generate a variable called “any kinds of male sex partners.” First, we asked whether they had regular and casual male sex partners in the past 3 months and whether they had unprotected anal intercourse with their male sex partners. Once they had male sex partners, we asked them how they requested the HIV serostatus of their male sex partners, including “I didn’t request,” “I requested orally or by message,” “I requested by taking HIV test together,” “I requested by sending my own HIV e-report,” and “I requested by asking for partner’s HIV e-report” while HIV e-report was available at the month 3 follow-up. If participants request at the month 3 follow-up, then they were asked how they received the HIV serostatus of their male sex partners by checking the response options (1=I did not receive partner’s disclosure, 2=I received partner’s disclosure orally or by message [namely, without any evidence], 3=I received partner’s disclosure by HIV reports or HIV test kits, and 4=I received partner’s disclosure by HIV e-reports).

#### HIV e-Reports

Participants were asked whether they had HIV e-reports, whether they received others’ HIV e-reports, and whether they sent HIV e-reports to others in the past 3 months. HIV e-report has been operating in Guangzhou after the midyear of 2019 when the baseline of this study was initiated. HIV e-reports–related results were only available at the month 3 follow-up.

### Statistical Analysis

Descriptive statistics were used to depict participants’ sociodemographic characteristics, MSM-related information, HIV-related information, HIV serostatus request, and disclosure receiving from different kinds of male sex partners.

A percentage stacked bar graph was used to describe the manner of HIV serostatus request at baseline and the month 3 follow-up. Another bar graph was used to depict the manner of receiving HIV serostatus disclosures at the month 3 follow-up.

Univariate associations were assessed using binary logistic regression to examine each of the independent variables listed above with the 2 outcomes of HIV serostatus request behavior, which were “Had request the HIV serostatus of any kinds of male sex partners at the month 3 follow-up?” and response behaviors to HIV serostatus request, “Had received HIV serostatus disclosure from any kinds of male sex partners at the month 3 follow-up?” Subsequently, significant variables (*P*<.05) from the univariate logistic regression analysis were included in the multivariate logistic regression analysis. Multivariate stepwise logistic regression was applied to select the final model.

Measures of association were presented as univariate odds ratio versus multivariate odds ratio (OR_m_), with 95% CI. All statistical analyses were performed using R (version 4.2.1) with 2-tailed test. A *P*<.05 was considered statistically significant. The packages used were dplyr, tableone, ggplot, and glm.

### Ethical Considerations

The study protocol was approved by the Ethics Committee of Sun Yat-sen University (Institutional Review Board number 054/19; February 28, 2019). Informed consent was obtained from each ego at the clinic and from each alter on the internet through the WeChat-based system. The study data were collected and stored securely on the servers of the Guangzhou CDC HIV testing service system, WeChat-based database, and web-based questionnaire database, in accordance with relevant data protection laws and regulations. Access to these databases was restricted to the research team in order to ensure the confidentiality and privacy of the data.

## Results

### Recruitment

From September 2019 to August 2020, a total of 1607 MSM who took HIV testing at the Lingnan Center were invited to participate in this study. Of these, 1295 were recruited as Egos. Egos subsequently sent HIV e-reports to 1782 contacts via WeChat, and 702 of them (response rate, 22.3%, 702/1295) clicked the link to the questionnaire. At baseline, 397 participants were recruited as alters. Of these, 40 were lost at the month 3 follow-up whose HIV e-report information and HIV serostatus disclosure behavior information were missing. As a result, a total of 357 participants were included in the final analysis.

### Characteristics of Participants

Out of 357 participants, around half of them were over 25 years of age (195/357, 54.6%), educated above high school (200/357, 56%), and earned more than 5000 RMB (US $700) per month (198/357, 55.5%). Most of them were not married to women (339/357, 95%).

Most participants were homosexual (295/357, 82.6%) and the proportion of self-identified sex role was approximately similar (122/357, 34.2% in insertive; 126/357, 35.3% in receptive; and 109/357, 30.5% in both).

A total of 79% (282/357) of participants took part in HIV-related programs in the past 3 months. HIV stigma scores ranged from8 to 23 and were at a high level (median 19, IQR 17-22) overall. On average, participants’ social norm (median 3, IQR 2.67-3) inclined to a positive direction. Further details on participants characteristics participants' characteristics were presented in Table 1.

**Table 1:** Background characteristics of alters who enrolled at the first project year from Sep 2019 to Aug 2020.

					Alters (N=357), n (%)
**Sociodemographic characteristics**					
	**Age (years)**				
		≤25 years		162 (45.4)	
		>25 years		195 (54.6)	
	**Currently married to a woman**				
		No		339 (95)	
		Yes		18 (5)	
	**Guangzhou permanent resident (Hukou)**				
		No		106 (29.7)	
		Yes		251 (70.3)	
	**How long had been in Guangzhou**				
		≤3 years		151 (42.3)	
		>3 years		206 (57.7)	
	**Highest education obtained**				
		High school or below		157 (44)	
		Above high school		200 (56)	
	**Personal monthly income (5000 RMB=US $700)**				
		≤5000		159 (44.5)	
		>5000		198 (55.5)	
**MSM^a^-related characteristics**					
	**Self-identified sex orientation**				
			Bisexual, heterosexual, or not sure		62 (17.4)
			Homosexual		295 (82.6)
	**Self-identified sex role**				
			Insertive only		122 (34.2)
			Receptive only		126 (35.3)
			Both		109 (30.5)
	**Male sex partner mostly recruited from the internet**				
			No		13 (3.6)
			Yes		344 (96.4)
**HIV-related information^b^**					
	**Had intervened in any HIV-related programs in the past** **3** **months**				
			No		75 (21)
			Yes		282 (79)
	**Had taken up HIV antibody testing in the past** **3** **months**				
			No		182 (51)
			Yes		175 (49)
	**Had drug use during sex in the past** **3** **months**				
			No		279 (78.2)
			Yes		78 (21.8)
	**How do you think the prevalence of HIV infection in MSM in Guangzhou**				
			<10%		79 (22.1)
			≥10%		278 (77.9)
	**Is someone close to you infected with HIV**				
			No		226 (63.3)
			Yes		131 (36.7)

^a^MSM: men who have sex with men.

^b^Median HIV stigma score 19 (IQR 17-22) and median HIV testing social norm score 3 (IQR 2.67-3.00).

### HIV e-Reports Emerging as the New Approach for HIV Serostatus Requests and Disclosure Receiving

**Figure 3:**
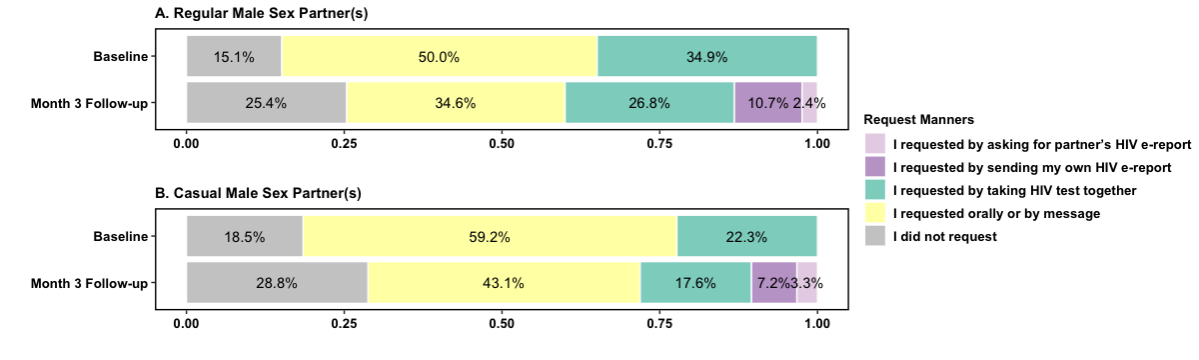
The proportion of MSM’s HIV serostatus requesting manners in different types of male sex partners at baseline and the month 3 follow-up. MSM: men who have sex with men.

At baseline, HIV serostatus request behaviors contained only 3 types of manners, including “I did not request,” “I requested orally or by message,” and “I requested by taking HIV test together.” The proportions for requesting manners were 15.1% (35/232), 50.0% (116/232), and 34.9% (81/232) in 232 participants with regular male sex partners and were 18.5% (34/184), 59.2% (109/184), and 22.3% (41/184) in 184 participants with casual male sex partners, respectively (Figure 3).

At month 3 follow-up, for all 357 participants, 57.4% (205/357) of them had regular male sex partners, 42.9% (153/357) of them had casual male sex partners, and 73.4% (262/357) of them had either kind of male sex partner in the past 3 months.

After HIV e-reports were available, out of all 357 participants, 32.2% (n=115) of them had their own e-reports, and 37.8% (n=135) of them had received others’ HIV e-reports at month 3 follow-up. For those who had their own HIV e-reports, 50.4% (58/115) of them had sent their own HIV e-reports to others. Therefore, 2 new request approaches for HIV serostatus using the HIV e-report emerged; namely “I requested by sending my own HIV e-report” and “I requested by asking for partner’s HIV e-report.” The proportions of these 2 approaches were 10.7% (22/205) and 2.4% (5/205) toward regular male sex partners, and 7.2% (11/153) and 3.3% (5/153) toward casual male sex partners, respectively. See Figure 3 and Table 2.

**Figure 4:**
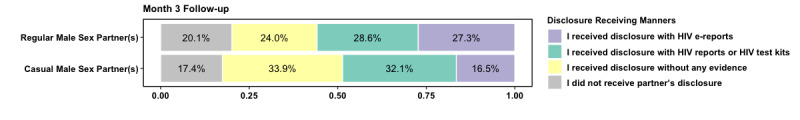
The proportion of MSM’s HIV serostatus disclosure receiving manners in different types of male sex partners at the month 3 follow-up. MSM: men who have sex with men.

Participants’ casual sex partners were more likely to disclose HIV serostatus without any evidence (37/109, 33.9%) and with HIV regular reports or HIV test kits (35/109, 32.1%), compared with their regular sex partners (37/158, 24% and 44/158, 28.6%). Instead, participants were more likely to receive HIV serostatus disclosure via e-reports from regular male sex partners (42/158, 27.3%) than casual male sex partners (18/109, 16.5%; Figure 4).

**Table 2:** HIV serostatus request and disclosure receiving behaviors toward different male sex partners among alters at month 3 follow-up.

			Participants (N=357), n (%)
**Regular male sex partner information in the past 3 months**			
	**Whether had regular male sex partners**		
		No	152 (42.6)
		Yes	205 (57.4)
	**Whether had had UAI^a^ with regular male sex partners**		
		No	60 (29.3)
		Yes	145 (70.7)
	**How did you request the HIV serostatus from regular male sex partners**		
		I did not request	51 (24.9)
		I requested orally or by message	72 (35.1)
		I requested by taking HIV test together	55 (26.8)
		I requested by sending my own HIV e-report	22 (10.8)
		I requested by asking for partner’s HIV e-report	5 (2.4)
	**How did you receive disclosure of the HIV serostatus from regular male sex partners**		
		I did not receive partner’s disclosure	31 (20.1)
		I received disclosure without any evidence	37 (24)
		I received disclosure with HIV reports or HIV test kits	44 (28.6)
		I received disclosure with HIV e-reports	42 (27.3)
**Casual male sex partner information in the past 3 months**			
	**Whether had casual male sex partners**		
		No	204 (57.1)
		Yes	153 (42.9)
	**Whether had had UAI with casual male sex partners**		
		No	29 (19)
		Yes	124 (81)
	**How did you request the HIV serostatus of casual male sex partners**		
		I did not request	44 (28.8)
		I requested orally or by message	66 (43.1)
		I requested by taking HIV test together	27 (17.6)
		I requested by sending my own HIV e-report	11 (7.2)
		I requested by asking for partner’s HIV e-report	5 (3.3)
	**How did you receive disclosure of the HIV serostatus from casual male sex partners**		
		I did not receive partner’s disclosure	44 (32.8)
		I received disclosure without any evidence	37 (27.6)
		I received disclosure with HIV reports or HIV test kits	35 (26.2)
		I received disclosure with HIV e-reports	18 (13.4)
**Any kinds of male sex partner information in the past 3 months**			
	**Whether had any kinds of male sex partners**		
		No	95 (26.6)
		Yes	262 (73.4)
	**Whether had UAI with any male sex partners**		
		No	66 (25.2)
		Yes	196 (74.8)
	**How did you request the HIV serostatus of any kinds of male sex partners**		
		I did not request	67 (25.6)
		I requested orally, by message, or by taking HIV test together	160 (61)
		I requested by HIV e-reports	35 (13.4)
	**How did you receive disclosure of the HIV serostatus from any kinds of male sex partners**		
		I did not receive partner’s disclosure	32 (16.4)
		I received disclosure with no evidence, HIV reports, or HIV test kits	112 (57.4)
		I received disclosure with HIV e-reports	51 (26.2)
**HIV e-reports–related information in the past 3 months**			
	**Had sent your own HIV e-reports to others**		
		I didn’t have my own HIV e-reports	242 (67.8)
		I had my own HIV e-reports but didn’t send to others^b^	57 (16)
		I had my own HIV e-reports and sent to others^b^	58 (16.2)
	**Had received others’ HIV e-reports^c^**		
		No	222 (62.2)
		Yes	135 (37.8)

^a^UAI: unprotected anal intercourse.

^b^Had their own e-reports at the month 3 follow-up.

^c^Despite the HIV e-report which ego sent to alter for recruitment.

### Factors Associated With HIV Serostatus Requests and Receiving Disclosures

In univariate logistic analysis, 5 factors were significantly (*P*<.05) associated with HIV serostatus request behavior. Further details were described in Table 3.

The multivariate stepwise regression model showed that HIV e-report–related variables, namely, having had HIV e-reports but didn’t send to others (OR_m_ 2.71, 95% CI 1.19-6.86; *P*=.02; reference: participants who didn’t have their own HIV e-reports), having had HIV e-reports and sent to others (OR_m_ 2.67, 95% CI 1.07-7.73; *P*=.048; reference: participants who didn’t have their own HIV e-reports), and having had received others’ HIV e-reports (OR_m_ 2.03, 95% CI 1.04-4.11, *P*=.04) were significantly associated with HIV serostatus request behavior. For variables about HIV-related information in the past 3 months, namely higher HIV testing behavior social norm scores (OR_m_ 2.13, 95% CI 1.12-4.17; *P*=.02), having had intervened by any HIV-related programs (OR_m_ 2.28, 95% CI 1.12-4.65; *P*=.02), and having taken up HIV antibody testing (OR_m_ 1.85, 95% CI 1.00-3.44; *P*=.05) in the past 3 months, remained in the final model as well.

All variables listed in Table 2 were not associated with receiving HIV serostatus disclosures (not tabulated).

**Table 3:** Univariate and multivariate regression analysis of associated factors with HIV serostatus request behavior. Univariate logistic regressions were conducted for “Had request the HIV serostatus of any kinds of male sex partners at the month 3 follow-up” and “Had any kinds of male sex partners inform you the HIV serostatus at the month 3 follow-up.” All variables in were contained in univariate logistic regressions, and those with *P* value of >.10 were excluded. No factor significantly associated with “Had any kinds of male sex partners inform you the HIV serostatus at the month 3 follow-up.”

		All, N	Participants, n (%)	Had requested HIV serostatus^a^ from any kinds of male sex partners at the month 3 follow-up	
				OR^b^ (95% CI)	OR_m_^c^ (95% CI)
**Had sent your own HIV e-reports to other MSM in the past 3 months**					
	I hadn’t had my own HIV e-reports	165	112 (67.9)	Reference	Reference
	I had had my own HIV e-reports but hadn’t sent to others	47	39 (83)	2.31 (1.05-5.63)	2.71 (1.19-6.86)
	I had had my own HIV e-reports and had sent to others	50	44 (88)	3.47 (1.49-9.53)	2.67 (1.07-7.73)
**Had received other MSM’s HIV e-reports in the past 3 months**					
	No	164	112 (68.3)	reference	reference
	Yes	98	83 (85.7)	2.57 (1.35-4.88)	2.03 (1.04-4.11)
**Had taken up HIV antibody testing in the past 3 months**					
	No	120	79 (65.8)	Reference	reference
	Yes	142	116 (81.7)	2.32 (1.31-4.09)	1.85 (1.00-3.44)
**Had intervened in any HIV-related programs in the past 3 months**					
	No	51	31 (60.8)	Reference	reference
	Yes	211	164 (77.7)	2.25 (1.18-4.31)	2.28 (1.12-4.65)

^a^Median HIV testing behavior social norm score was 3 (IQR 2.67-3.00) overall, while that for participants who had requested HIV serostatus from any kinds of male sex partners at month 3 follow-up was 1.5 (95% CI 1.03-1.28) (ie, OR group) and 2.13 (95% CI 1.12-4.17) (ie, OR_m_ group).

^b^OR: univariate odds ratio.

^c^OR_m_: multivariate odds ratio. Five variables were put in the multivariate model, and Akaike’s information criterion was used to select the variables in this model.

## Discussion

### Principal Results

e-Reports are a new approach for HIV serostatus request and disclosure for the HIV risk population. MSM chose HIV e-report as theweb-based approach to disclose their own HIV serostatus or to request partner’s HIV serostatus with authenticity when it was available in Guangzhou. The most important is that HIV serostatus request behaviors were positively associated with having their own HIV e-report. To the best of our knowledge, this is the first study to discuss the use of HIV e-reports and explore its association. HIV e-report, codeveloped by the MSM community itself, could be considered a novel approach to promote mutual HIV status disclosure before engaging in sexual behaviors among HIV high-risk population, and being capable to be replicated in other countries and regions based on the ability of building information platforms.

The most interesting finding is that MSM who used HIV e-reports were more likely to actively request the HIV serostatus of their male sex partners. As e-report is a new modality in the HIV research area, studies to investigatethe association between HIV e-reports and HIV serostatus request and disclosure behaviors have rarely been reported. The possible reason for this finding is the confidence in the e-report, which is the CDC-certified credible evidence of HIV serostatus that cannot be modified. Holding an HIV e-report may make MSM feel more confident and self-assured when requesting the HIV serostatus of their male sex partners. Therefore, HIV e-report has the potential to be applied as an intervention to control the risk of HIV infection by promoting HIV serostatus disclosure. However, it is important to emphasize that the HIV e-report is not a substitute for condom use. A meta-analysis conducted in 2017 showed that seroadaptive substitution for condoms can increase the risk of HIV infection [20]. This study demonstrates the favorable influence of HIV e-report on HIV-related behaviors in the MSM community in the short term, and the long-term impact of HIV e-report among HIV risk population is expected to be explored in the future.

After the HIV e-report was available, a new proportion of MSM had used the e-report to request their sex partner’s HIV serostatus (13.4%, 35/262) and had received their sex partner’s e-report as an HIV serostatus disclosure (26.2%, 51/195). In addition to e-report results certificated by CDC, the significance of HIV e-report is that it is driven by MSM community demand, which correspondingly engaged in the development of HIV e-report. MSM designed it because they feel sending out their own HIV e-report is the most natural and credible way to request male sex partners’ HIV serostatusas well as disclose their own HIV serostatus. According to the results, HIV e-report is becoming more acceptable within the MSM community.

We found that nearly one-third of MSM did not request the HIV serostatus of their casual male sex partners. A total of 74.9% (197/232) of alters requested their regular sex partner’s HIV serostatus, while 71.5% (150/184) of alters requested their casual sex partner’s HIV serostatus at baseline. Requesting a partner’s HIV serostatus before engaging in sexual activity is an active coping strategy to control the risk of HIV infection. However, most research on HIV serostatus focuses on self-disclosure rather than request behavior [21-25]. Only a few studies have investigated HIV serostatus request behavior, and our data contribute to the literature [5,6]. Some influence factors have been identified in studies, such as perceived high HIV risk, lower HIV-related stigma, and greater engagement with the MSM community [26,27]. HIV serostatus request, as a proactive behavior, which can elevate the importance of consciousness about HIV serostatus disclosure before sex, has ample room for improvement among MSM.

In our study, we found that 62.2% (163/262) of participants reported receiving disclosure from male sex partners. Other studies showed that the HIV serostatus self-disclosure rate among MSM in China was approximately 20% in 2016 and 2018 [22,28]. We hypothesize that this higher disclosure rate may be due to increased awareness of the importance of disclosure over the past 5 years. We did not identify any factors associated with receiving an HIV serostatus disclosure. The possible reason may be that receiving a disclosure is a passive behavior that is primarily influenced by the characteristics of the person who disclosed their status rather than the recipient. Furthermore, our study only collected information from the recipient’s perspective, which limits our ability to identify factors that may influence disclosure receiving behavior. Active coping strategies used by MSM, such as promoting the active behavior of requests by expanding the use of HIV e-reports, should be promoted.

Finally, previous studies indicated that HIV testing is positively associated with HIV serostatus request and disclosure [22,27], and our study found corroborating results. Furthermore, MSM who had higher HIV testing social norm scores and had intervened in any HIV-related programs were more likely to request HIV serostatus. This suggests that promoting both health education and HIV serostatus request and disclosure synchronously could be a promising route.

### Limitations

This study is subject to several limitations. First, as the baseline and month 3 follow-up questionnaires collected alters’ information in the past 3 months, the information and recall bias might exist as other HIV-related publications [29,30]. Second, there might be selection bias since participants were recruited through HIV testers from a local MSM-friendly clinic in Guangzhou and questionnaires were conducted on the mobile app, which led to participants being younger and well educated. Third, this is an observational study. Though we found several factors associated with HIV serostatus request, the causation between them needs further study.

### Conclusions

This study indicated that the HIV e-report, the health service tool coproduced by community members, has become acceptable and could be used as a new optional approach for HIV serostatus request and disclosure among populations at high risk of sexually transmitted infections. In particular, the use of HIV e-report has potential influence on promoting HIV serostatus request behaviors. It is anticipated that the e-report approach will have an extended spectrum of coverage to reach more target populations and ultimately accelerate the decline of infectious disease transmission.

## Abbreviations

CDC: Center for Disease Control and Prevention
MSM: men who have sex with men
ORm: multivariate odds ratio
