# Correction: WeChat-Based HIV e-Report, a New Approach for HIV Serostatus Requests and Disclosures Among Men Who Have Sex With Men: Prospective Subgroup Analysis of a Randomized Controlled Trial

**DOI:** 10.2196/48961

**Published:** 2023-05-17

**Authors:** Hai-Tong Sun, Xiao-Ru Fan, Yu-Zhou Gu, Yong-Heng Lu, Jia-Ling Qiu, Qing-Ling Yang, Jing-Hua Li, Jing Gu, Chun Hao

**Affiliations:** 1 Department of Medical Statistics, School of Public Health, Sun Yat-Sen University Guangzhou China; 2 Sun Yat‑Sen Global Health Institute, Institute of State Governance, Sun Yat-Sen University Guangzhou China; 3 Guangzhou Center for Disease Control and Prevention Guangzhou China; 4 Lingnan Community Support Center Guangzhou China

In “WeChat-Based HIV e-Report, a New Approach for HIV Serostatus Requests and Disclosures Among Men Who Have Sex With Men: Prospective Subgroup Analysis of a Randomized Controlled Trial” (JMIR mHealth and uHealth 2023;11:e48961), the following errors were corrected:

1. In the originally published article, the title appeared as follows:

WeChat-Based HIV e-Report, a New Manner for HIV Serostatus Request and Disclosure and Their Associated Factors Among Men Who Have Sex With Men: Prospective Subgroup Analysis of Randomized Controlled Trails

This has been corrected to:

WeChat-Based HIV e-Report, a New Approach for HIV Serostatus Requests and Disclosures Among Men Who Have Sex With Men: Prospective Subgroup Analysis of a Randomized Controlled Trial

2. In the Abstract: Background section of the originally published article, the second sentence appeared as follows:

However, the reliability of common methods for HIV serostatus requests and disclosure is unsatisfactory.

This has been corrected to:

However, the reliability of common methods for HIV serostatus request and disclosure is inadequate.

3. In the Abstract: Objectives section of the originally published article, the second sentence appeared as follows:

Additionally, the study aimed to explore its correlates with HIV serostatus requesting and disclosure receiving behavior.

This has been corrected to:

Additionally, the study aimed to explore its correlation with HIV serostatus requesting and disclosure receiving behavior.

4. In the Abstract: Methods section of the originally published article, the third sentence appeared as follows:

Participants completed web-based questionnaires at baseline and at the month 3 follow-up, which covered sociodemographic characteristics, HIV-related information, HIV serostatus requests, HIV serostatus disclosure receiving, and HIV e-report usage.

This has been corrected to:

Participants completed web-based questionnaires at baseline and at the month 3 follow-up, which covered sociodemographic characteristics, HIV-related information, HIV serostatus requests, receiving HIV serostatus disclosures, and HIV e-report usage.

5. In the Abstract: Results section of the originally published article, the second sentence appeared as follows:

For HIV serostatus requests, 13.1% (27/205) and 10.5% (16/153) of participants started to use HIV e-reports to ask the HIV serostatus of regular and casual male sex partners, respectively. Of the regular and casual male sex partners, 27.3% (42/154) and 16.5% (18/109), respectively, chose HIV e-reports to disclose HIV serostatus. Compared to MSM who did not have HIV e-reports, those who said

…

This has been corrected to:

In all, 13.1% (27/205) and 10.5% (16/153) of participants started to use HIV e-reports to request the HIV serostatus from regular and casual male sex partners, respectively. Moreover, 27.3% (42/154) and 16.5% (18/109) of the regular and casual male sex partners, respectively, chose HIV e-reports to disclose their HIV serostatus. Compared to MSM who did not have HIV e-reports, those who had HIV e-reports and stated [...]

6. In the Abstract: Results section of the originally published article, the last sentence appeared as follows:

Whereas no factor was associated with HIV serostatus disclosure received from partners.

This has been corrected to:

However, no factor was associated with receiving an HIV serostatus disclosure from partners.

7. In the Abstract: Conclusions section of the originally published article, the first sentence appeared as follows:

The HIV e-report has been accepted by the MSM community in Guangzhou and could be applied as a new optional way for HIV serostatus request and disclosure.

This has been corrected to:

The HIV e-report has been accepted by the MSM community in Guangzhou and could be applied as a new optional approach for HIV serostatus requests and disclosures.

8. In the Introduction section of the originally published article, the second paragraph appeared as follows:

Studies indicated that over half of MSM used verbal communication and guessing for HIV serostatus requesting and disclosing among MSM [5, 6]. While taking HIV tests together is a reliable manner to confirm partners’ HIV status, some individuals doubt the reliability of HIV self-testing, and self-test kits may not always be readily available. Deception of HIV serostatus in verbal information is prevalent [7] and difficult to confirm.

This has been corrected to:

Studies indicated that over half of MSM used verbal communication and guessing for HIV serostatus request and disclosure among MSM [5,6]. While taking HIV tests together is a reliable approach to confirm partners’ HIV status, some individuals doubt the reliability of HIV self-testing, and self-test kits may not always be readily available. Deception of HIV serostatus in verbal information is prevalent [7] and difficult to confirm.

9. In the Introduction section of the originally published article, the third paragraph appeared as follows:

WeChat, a popular social media app with over 1.2 billion active users [8], similar to Twitter or the mix of WhatsApp and Facebook, is an ubiquitous daily use app in China [9]. WeChat miniprograms are subapps within the WeChat ecosystem. It has great potential for health intervention research [10]. In Guangzhou city, a unique and well-established WeChat miniprogram of the HIV testing service system in China is developed by Guangzhou Centers for Disease Control and Prevention (CDC) and MSM community-based organization Lingnan Partners Community Support Center (hereinafter called “Lingnan Center”) [11].

This has been corrected to:

WeChat, a popular social media app with over 1.2 billion active users [8], similar to Twitter or the mix of WhatsApp and Facebook, isa ubiquitous daily-use appin China [9]. WeChat miniprograms are subapps within the WeChat ecosystem. It has great potential for health intervention research [10]. In Guangzhou city, a unique and well-established WeChat miniprogram of the HIV testing service system in China has been developed by Guangzhou Centers for Disease Control and Prevention (CDC) and the MSM community-based organization Lingnan Partners Community Support Center (hereinafter called “Lingnan Center”) [11].

10. In the Introduction section of the originally published article, the last paragraph appears as follows:

The objective of this study is to describe the usage of the HIV e-report after it was available in Guangzhou and investigate whether it is associated with promoting HIV serostatus requests, and disclosure-related behaviors among this high-risk population.

This has been corrected to:

The objective of this study is to describe the usage of the HIV e-report after it was available in Guangzhou and investigate whether it is associated with promoting HIV serostatus requests and disclosure-related behaviors among this high-risk population.

11. In the Methods: Recruitment of Participants section of the originally published article, the second sentence of the third paragraph appears as follows:

After 3 months, alters participants would receive WeChat messages which contain the link to the follow-up questionnaire.

This has been corrected as follows.

After 3 months, alters would receive WeChat messages which contain the link to the follow-up questionnaire.

12. In the Methods: Measures: Sociodemographic Characteristics section of the originally published article, the first sentence appeared as follows:

All background characteristics of alter participants were collected in the baseline questionnaire.

This has been corrected as follows:

All background characteristics of alters were collected in the baseline questionnaire.

13. In the Methods: Measures: Sociodemographic Characteristics section of the originally published article, the last sentence appeared as follows:

We dichotomize age by 25 years, income by 5000 RMB (US $700) according to median.

This has been corrected as follows:

We dichotomized age by 25 years and income by 5000 RMB (US $700) according to the median.

14. In the Methods: Statistical Analysis section of the originally published article, the last sentence of the second paragraph appeared as follows:

Another bar graph was used to depict the manner of HIV serostatus disclosure receiving at the month 3 follow-up.

This has been corrected as follows:

Another bar graph was used to depict the manner of receiving HIV serostatus disclosures at the month 3 follow-up.

15. In the Results: Characteristics of Participants section of the originally published article, the third paragraph appeared as follows:

A total of 79% (282/357) of participants had intervened by HIV-related programs in the past 3 months. HIV stigma scores ranged from 8 to 23, and at a high level (median 19, IQR 17-22) overall. On average, participants’ social norm (median 3, IQR 2.67-3) inclined to a positive direction. Further details on participants characteristics were presented in Table 1.

This has been corrected as follows:

A total of 79% (282/357) of participants took part in HIV-related programs in the past 3 months. HIV stigma scores ranged from8 to 23 and were at a high level (median 19, IQR 17-22) overall. On average, participants’ social norm (median 3, IQR 2.67-3) inclined to a positive direction. Further details on participants' characteristics were presented in Table 1.

16. In the Results: HIV e-Reports Emerging as the New Approach for HIV Serostatus Requests and Disclosure Receiving section of the originally published article, the section subtitle appeared as follows:

HIV e-Reports Emerging as the New Manner of HIV Serostatus Request and Disclosure Receiving

This has been corrected as follows:

HIV e-Reports Emerging as the New Approach for HIV Serostatus Requests and Disclosure Receiving

17. In the Results: HIV e-Reports Emerging as the New Approach for HIV Serostatus Requests and Disclosure Receiving section of the originally published article, the second paragraph appeared as follows:

At month 3 follow-up, for all 357 participants, 57.4% (205/357) of them had regular male sex partners, 42.9% (153/357) of them had casual male sex partners, and 73.4% (262/357) of them had any kind of male sex partners in the past 3 months.

This has been corrected as follows:

At month 3 follow-up, for all 357 participants, 57.4% (205/357) of them had regular male sex partners, 42.9% (153/357) of them had casual male sex partners, and 73.4% (262/357) of them had either kind of male sex partner in the past 3 months.

18. In the Results: HIV e-Reports Emerging as the New Approach for HIV Serostatus Requests and Disclosure Receiving section of the originally published article, the third sentence of the third paragraph appeared as follows:

Therefore, 2 new request ways for HIV serostatus using the HIV e-report emerged; namely “I requested by sending my own HIV e-report” and “I requested by asking for partner’s HIV e-report.”

This has been corrected as follows:

Therefore, 2 new request approaches for HIV serostatus using the HIV e-report emerged; namely “I requested by sending my own HIV e-report” and “I requested by asking for partner’s HIV e-report.”

19. In the Results: HIV e-Reports Emerging as the New Approach for HIV Serostatus Requests and Disclosure Receiving section of the originally published article, the fourth sentence of the third paragraph appeared as follows:

The proportions of these 2 ways were 10.7% (22/205) and 2.4% (5/205) toward regular male sex partners, and 7.2% (11/153) and 3.3% (5/153) toward casual male sex partners, respectively.

This has been corrected as follows:

The proportions of these 2 approaches were 10.7% (22/205) and 2.4% (5/205) toward regular male sex partners, and 7.2% (11/153) and 3.3% (5/153) toward casual male sex partners, respectively.

20. In the Methods: HIV e-Reports Emerging as the New Approach for HIV Serostatus Requests and Disclosure Receiving section of the originally published article, the title of Table 2 appeared as follows:

Table 2. HIV serostatus request and disclosure receiving behaviors from different male sex partners among alters at month 3 follow-up.

This has been corrected as follows:

Table 2. HIV serostatus request and disclosure receiving behaviors toward different male sex partners among alters at month 3 follow-up.

21. In the Results: Factors Associated With HIV Serostatus Requests and Receiving Disclosure section of the originally published article, the subsection title appeared as follows:  

Associated Factors With HIV Serostatus Request and Disclosure Receiving

This has been corrected as follows:

Factors Associated With HIV Serostatus Requests and Receiving Disclosures

22. In the Factors Associated With HIV Serostatus Requests and Receiving Disclosure section of the originally published article, the last paragraph appeared as follows:

All variables listed in Table 2 were not associated with HIV serostatus disclosure receiving (not tabulated).

This has been corrected as follows:

All variables listed in Table 2 were not associated with receiving HIV serostatus disclosures (not tabulated).

23. In the Discussion: Principal Results section of the originally published article, the first two sentences appeared as follows:

e-Report is emerging as a new manner for HIV serostatus request and disclosure for the HIV risk population. MSM chose HIV e-report as the web-based way to disclose their own HIV serostatus or to request partner’s HIV serostatus with authenticity when it was available in Guangzhou.

This has been corrected as follows:

e-Reports are a new approach for HIV serostatus request and disclosure for the HIV risk population. MSM chose HIV e-report as theweb-based approach to disclose their own HIV serostatus or to request partner’s HIV serostatus with authenticity when it was available in Guangzhou.

24. In the Discussion: Principal Results section of the originally published article, the fourth sentence appeared as follows:

To the best of our knowledge, this is the first study that mentioned the HIV e-report and explored its association with HIV disclosure–related behaviors.

This has been corrected as follows:

To the best of our knowledge, this is the first study to discuss the use of HIV e-reports and explore its association.

25. In the Discussion: Principal Results section of the originally published article, the fifth sentence appeared as follows:

HIV e-report, codeveloped by MSM community itself, could be considered a novel approach to promote mutual HIV status disclosure before engaging in sexual behaviors among HIV high-risk population, and being capable to be replicated in other countries and regions based on the ability of building information platforms.

This has been corrected as follows:

HIV e-report, codeveloped by the MSM community itself, could be considered a novel approach to promote mutual HIV status disclosure before engaging in sexual behaviors among HIV high-risk population, and being capable to be replicated in other countries and regions based on the ability of building information platforms.

26. In the Discussion: Principal Results section of the originally published article, the second sentence of the second paragraph appeared as follows:

As e-report is a new modality in the HIV research area, studies to investigate the association between HIV e-report, HIV serostatus request, and disclosure behaviors have rarely been reported.

This has been corrected as follows:

As e-report is a new modality in the HIV research area, studies to investigatethe association between HIV e-reports and HIV serostatus request and disclosure behaviors have rarely been reported.

27. In the Discussion: Principal Results section of the originally published article, the sixth sentence of the second paragraph appeared as follows:

However, it is important to emphasize that HIV e-report is not a substitute for condom use.

This has been corrected as follows:

However, it is important to emphasize that the HIV e-report is not a substitute for condom use.

28. In the Discussion: Principal Results section of the originally published article, the first sentence of the third paragraph appeared as follows:

After HIV e-report was available, a new portion of MSM had applied e-report to request sex partner’s HIV serostatus (13.4%, 35/262) and had received sex partner’s e-report as HIV serostatus disclosure (26.2%, 51/195).

This has been corrected as follows:

After the HIV e-report was available, a new proportion of MSM had used the e-report to request their sex partner’s HIV serostatus (13.4%, 35/262) and had received their sex partner’s e-report as an HIV serostatus disclosure (26.2%, 51/195).

29. In the Discussion: Principal Results section of the originally published article, the third sentence of the third paragraph appeared as follows:

MSM designed it because they feel sending out their own HIV e-report is the most natural and credible way to request male sex partners’ HIV serostatus as well as disclosure of their own HIV serostatus.

This has been corrected as follows:

MSM designed it because they feel sending out their own HIV e-report is the most natural and credible way to request male sex partners’ HIV serostatusas well as disclose their own HIV serostatus.

30. In the Discussion: Principal Results section of the originally published article, the fifth sentence of the fourth paragraph appeared as follows:

Only a few studies have investigated HIV serostatus request behavior, and our finding data contribute to the literature [5,6].

This has been corrected as follows:

Only a few studies have investigated HIV serostatus request behavior, and our data contribute to the literature [5,6].

31. In the Discussion: Principal Results section of the originally published article, the fourth sentence of the fifth paragraph appeared as follows:

We did not identify any factors associated with HIV serostatus disclosure receiving.

This has been corrected as follows:

We did not identify any factors associated with receiving an HIV serostatus disclosure.

32. In the Discussion: Principal Results section of the originally published article, the fourth sentence of the sixth paragraph appeared as follows:

The possible reason may be that disclosure receiving is a passive behavior that is primarily influenced by the characteristics of the person who disclosed their status rather than the recipient.

This has been corrected as follows:

The possible reason may be that receiving a disclosure is a passive behavior that is primarily influenced by the characteristics of the person who disclosed their status rather than the recipient.

33. In the Discussion: Principal Results section of the originally published article, the last sentence of the sixth paragraph appeared as follows:

Active coping strategies toward MSM their own, such as promoting the active behaviors of request through expanding the use of HIV e-report, should be promoted.

This has been corrected as follows:

Active coping strategies used by MSM, such as promoting the active behavior of requests by expanding the use of HIV e-reports, should be promoted.

34. In the Discussion: Limitations section of the originally published article, the third sentence appeared as follows:

Second, there might be selection bias since participants were recruited through HIV testers from a local MSM-friendly clinic in Guangzhou and questionnaires were conducted on the mobile app, which led participants trend to be young and well-educated.

This has been corrected as follows:

Second, there might be selection bias since participants were recruited through HIV testers from a local MSM-friendly clinic in Guangzhou and questionnaires were conducted on the mobile app, which led to participants being younger and well educated.

35. In the Discussion: Limitations section of the originally published article, the last sentence appeared as follows:

Though we find several factors associated with HIV serostatus request, the causation between them needs further study.

This has been corrected as follows:

Though we found several factors associated with HIV serostatus request, the causation between them needs further study.

36. In the Discussion: Conclusions section of the originally published article, the first sentence appeared as follows:

This study indicated that the HIV e-report, the health service tool coproduced by community members, has become acceptable and could be used as a new optional manner for HIV serostatus request and disclosure among sexually transmitted infections high-risk population.

This has been corrected as follows:

This study indicated that the HIV e-report, the health service tool coproduced by community members, has become acceptable and could be used as a new optional approach for HIV serostatus request and disclosure among populations at high risk of sexually transmitted infections.

37. In the Discussion: Conclusions section of the originally published article, the last sentence appeared as follows:

It is anticipated that e-report manner are able to have an extended spectrum of coverage to reach more target populations and ultimately accelerating the decline of infectious disease transmission.

This has been corrected as follows:

It is anticipated that the e-report approach will have an extended spectrum of coverage to reach more target populations and ultimately accelerate the decline of infectious disease transmission.

The correction will appear in the online version of the paper on the JMIR Publications website on May 17, 2023, together with the publication 

